# Migrants’ access to healthcare services within the European Union: a content analysis of policy documents in Ireland, Portugal and Spain

**DOI:** 10.1186/s12992-018-0373-6

**Published:** 2018-06-15

**Authors:** Céline Ledoux, Eva Pilot, Esperanza Diaz, Thomas Krafft

**Affiliations:** 1 Maastricht Centre for Global Health, Maastricht University, P.O. Box 616, 6200 MD Maastricht, The Netherlands; 2 Department of Global Public Health and Primary Care, University of Bergen, Bergen, Norway; 3 Norwegian Centre for Minority Health Research, Oslo, Norway

**Keywords:** Migration, European Union, Inclusive healthcare policy, Access to health services, Migrants’ health

## Abstract

**Background:**

The current migration flow into Europe is leading to a growing ethnically diverse population in many European countries. Now more than ever, those populations have different healthcare needs, languages, traditions, and previous level of care. This higher level of diversity is likely to increase health inequalities that might challenge healthcare systems if not addressed. In this context, this study aims at reviewing the policy framework for migrants’ access to healthcare in Spain, Portugal and Ireland, countries with a long history of immigration, to identify lessons to be learned for policies on migrants’ health.

**Methods:**

A content analysis of official policy documents was undertaken and the conceptual framework developed by Mladowsky was adapted to classify the actions indicated in the policies.

**Results:**

The content analysis revealed that the policy aim for all three analysed countries is the improvement of the health status of the immigrant population based on equity and equality principles. The main strategies are the adaptation of services through actions targeting patients and providers, such as the implementation of cultural mediators and trainings for health professionals.

**Conclusion:**

The three countries propose a great range of policies aiming at improving access to healthcare services for immigrants that can inspire other European countries currently welcoming refugees. Developing inclusive policies, however does not necessarily mean they will be implemented or felt on the ground. Inclusive policies are indeed under threat due to the economic and social crises and due to the respective nationalistic attitudes towards integration. The European Union is challenged to take a more proactive leadership and ensure that countries effectively implement inclusive actions to improve migrant’s access to health services.

## Background

In 2015, more than 1 million people arrived into the European Union by sea routes alone according to the UNHCR [1]. A similar number has applied for asylum in Europe, escaping conflict in their country and in search of better economic prospects.

With the number of displaced people across the world tripling in the last decade, the number of people attempting to reach Europe is not expected to slow down any time soon. This provokes concern among both the population and the leaders of European countries.

These increased numbers are inevitably going to put a strain on health systems. Beyond the numbers, the real challenge is the change in the composition of the European population due to this influx. Indeed, this demographic pressure leads to a growing ethnically diverse population in many European countries, which has more than ever different healthcare needs, languages, traditions, expectations and previous levels of care. This higher level of diversity is likely to increase health inequalities that might challenge healthcare systems if not addressed.

Health inequalities have been defined as “differences in opportunity for different population groups which result in, for example, unequal life chances, access to health services, nutritious food, adequate housing” [2]. Immigrants and ethnic minority groups generally experience a lower socio-economic status than nationals, the main explaining factor for ethnic-related health inequalities [3–5]. What remains poorly highlighted however are the inequalities in accessing health services, due to language, costs, location, information, and simply entitlement to receive healthcare, contributing to the “Exhausted Migrant Effect” [6]. Indeed, their poorer health status usually arises only after a certain time spent in the welcoming country. On arrival, migrants generally present a better health status than nationals and migrants who have lived in the country for a long time [6]. This “Healthy Migrant Effect” has several origins, such as the healthier and wealthier status of those engaging in the migration. Still, over time, their health status decreases below the health status of the nationals due to several factors, including lower access to healthcare services [6]. This “Healthy Migrant Effect” is nevertheless not invariable: as a matter of example, refugees having spent some time in camps usually experience high health deficits due to their living conditions. Therefore, overcoming health inequalities means improving the access, the quality, and the appropriateness of health services for immigrants and ethnic minority groups in Europe directly at the point of arrival.

Although meeting the needs of migrants and ethnic minorities is challenging due to the heterogeneity inside of the “migrant” category, there is a common statement of the right of everyone to the “enjoyment of the highest attainable standard of physical and mental health” across all member states [7]. This common statement, the growing migration flow into the European Union and the thrust towards harmonisation across the European countries is therefore a call for a development of policies aiming at improving migrants’ health, at both European and national levels. This is what has slowly occured since 2000, with the European States paying more and more attention to migrants health [8]. However, the economic crisis constrained national policies on the matter. Despite the concerns raised by the European Union as early as 2006 about migrants’ health and its fostering for the development of related policies, the austerity measures imposed to the Member States greatly impacted the provision of health care services for migrants [9]. Lately, the rise of the right-wing parties have put a further strain on it, causing great concerns. More than ever, there is a need for the European Union to step in and proactively engage in increasing awareness and support for the development of policies for migrants’ health at national level.

Two different kinds of health policies can be identified: the legislative policies, entitling health rights to immigrants, and the specific responses to this entitlement by the health system to make it appropriate and accessible for the migrant population [10]. Health policies can target either the users or the providers: Targeting users means for example improving health literacy and well adapted information, while targeting the providers means training the staff to improve their knowledge on culturally adapted healthcare [11]. These type of policies helps achieve equity in healthcare, meaning that people with different needs have access to what they need to achieve and maintain health and wellbeing. Equity in healthcare is a means to achieve more equality in health.

The general objective of this study is to undertake a content analysis of migrants’ health policies across three European countries: Ireland, Spain and Portugal. These countries have been chosen due to their status of “receiving countries” for at least 20 years. Only the health policies enriched in national and regional plans will be considered. The policies targeting social determinants of health and the policies on legal entitlements will not be considered.

## Methods

### Study design and country selection

A content analysis of health policies for immigrants at country level was performed using Walt et al’s definition. A health policy is the “courses of action (and inaction) that affect the set of institutions, organizations, services and funding arrangements of the health system” [12]. Provisions on legal entitlements were excluded. The study is limited to health policies enriched in national and regional plans.

To select the countries, the following criteria were applied: Being a country with immigration history, having developed specific health policies for migrants, having French, English, Spanish, or Portuguese as a national language, since these are the language spoken by the authors.

The countries chosen were Ireland, Portugal, and Spain which have been “receiving countries” for at least 20 years. After continuous radical changes in trends during the 19th and 20th centuries, Ireland definitively turned to be a receiving country in the mid-1990s with a dramatic increase in the number of asylum applicants and non-European immigration flows. More than half of the immigrants arriving in Ireland between 2001 and 2004 were indeed non-European immigrants [13]. Traditionally an emigration country, Spain is now one of the most important immigration countries in Europe. Since the middle of the 1980s despite the flux of immigrants starting to be more controlled with the settlement of restrictions, Spain has faced a continuous augmentation in the number of immigrants, especially in the last decade. Not only the number is growing, but there is also a diversification of origins. In 2008, 5,22 million foreigners were registered to the municipalities, representing 11,3 of the total population of 46,1 million [14]. Portugal became a country of immigration starting from the 1960s, with a straight increase from 1974 onwards, when the country started to receive migrants from the African Portuguese-Speaking Countries (PALOP). The immigration level intensified in the 80s, and in 2007 there were close to 450.000 documented foreign citizens in the country, representing 4,1% of the total population [15]. Although not reflected in those numbers, the presence of a high number of undocumented migrants should be noted for both Spain and Portugal, which according to estimates would appear to be tens of thousands of cases.

Due to the functioning of the Spanish health system, policies at the regional level should be included into the analysis. Indeed, Spain has a national health system (SNS, or Sistema Nacional de Salud) governed by both the Ministry of Health and the Departments of Health of the 17 autonomous communities which hold most of the competencies in health. Each autonomous community has its own health service, with some differences in the organization of the provision of services management, but sharing the basic features [16].

The Spanish autonomous regions of Madrid and Andalusia were chosen following these criteria: They are both major autonomous regions (respectively third and second in terms of population), both have an integration and a health plan including policies addressing migrants or ethnic minorities health, and they have different proportion of immigrant populations while being two major welcoming regions. In 2007, the proportion of foreigners represented 6,6% of the population in Andalusia and 14,1% in the Madrid region [16].

### Document search and selection

A search of official policy documents was performed, using the websites of the departments of health and immigration of the selected countries and the general website of the selected regions. The publication lists of those departments were manually screened and those having “health”, “immigration”, “integration” or “cultural diversity” where retained for analysis (see Table 1).

**Table 1:** National and regional policy plans analysed in the study

Country	Level	Health policies	Immigration or ethnic minority plan
Spain	National	Plan de Calidad para el SNS. (2010)	Plan Estratégico Ciudadanía e Integración (2011–2014)
	Andalusia	IV Plan Andaluz de Salud (2013–2020)	III Plan Integral para la Inmigración en Andalucía Horizonte 2016
	Comunidad de Madrid	Plan de promoción de la salud y prevención (2011–2013) Plan estratégico de salud mental (2010–2014)	III Plan de integración (2009–2012)
Portugal	National	Plano nacional de Saúde (2011–2016)	II Plano para a integração dos imigrantes (2010–2013)
Ireland	National	National Health Strategy (2011–2016) (National Intercultural Health Strategy 2007–2012) Primary care operational plan 2015	Equal Status Acts 2000 and 2004 Towards 2016 – Social Partnership Agreement (2006–2015) Building an inclusive society (2002–2007) National action plan for social inclusion (2007–2016) National Action Plan against Racism: Planning for Diversity (2005–2008)

Inclusion criteria for the country policies were: Official national or regional policy document from one of the selected countries related to migration and / or to health, integration or cultural diversity; published between 2000 and 2016.

### Data analysis

**Fig. 1:**
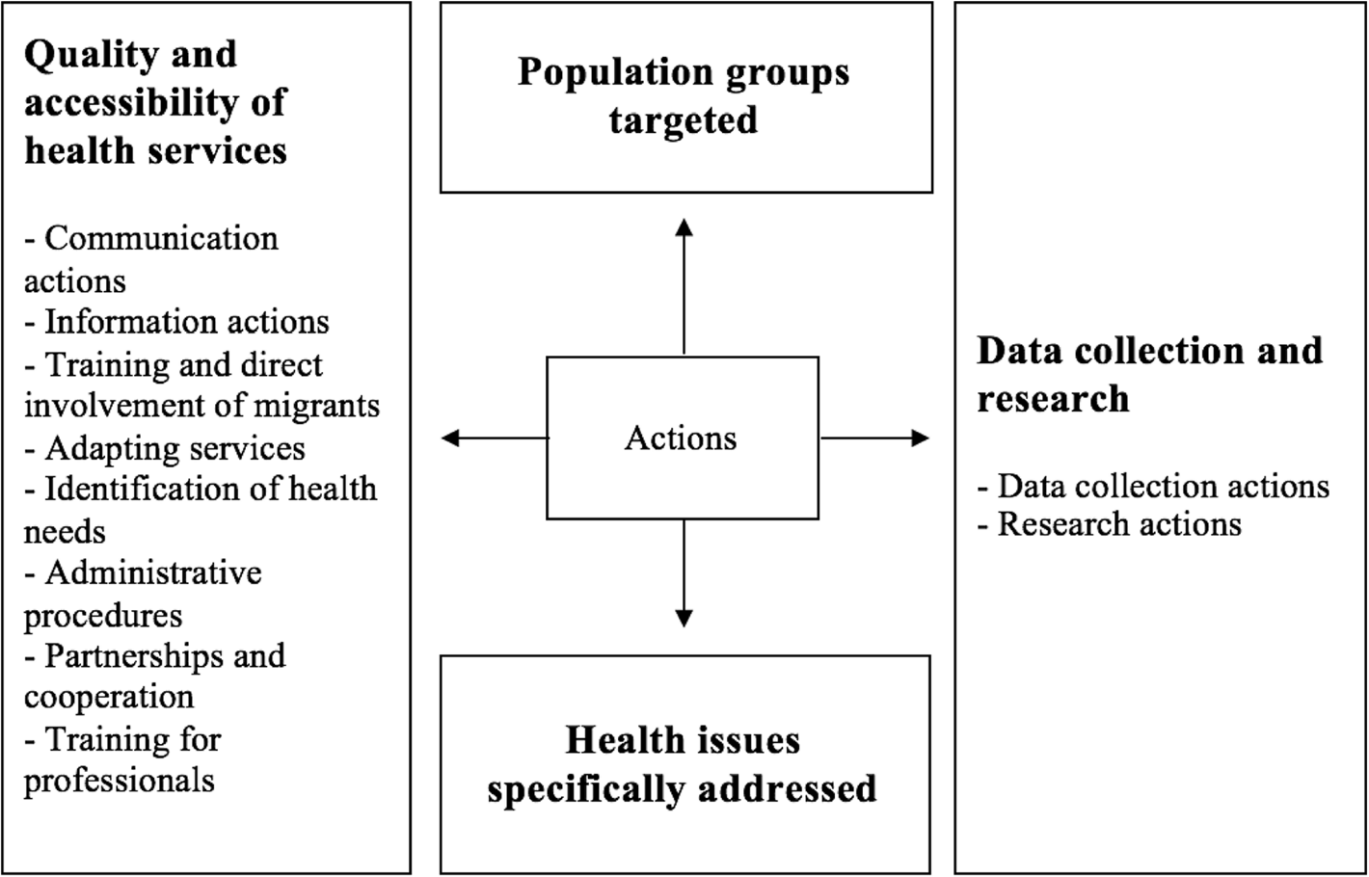
Conceptual framework Modified after Mladovsky conceptual framework [17]

For the country and regional health policy analysis, a content analysis was performed. The conceptual framework (Fig. 1) developed and proposed by Mladovsky [17] was adapted and used for the coding.

The first step consisted of reading the selected documents and marking the sections of the documents relevant to migrants health.

For the second stage consisting in the coding of the data, the original categories of the framework were slightly modified and used as pre-set codes. The actions listed in the policy documents were classified according to the following categories: (i) Population groups targeted; (ii) Data collection and research; (iii) Quality and accessibility of health services (iv) Health issues addressed.

Finally, the subcategories of the Mladovsky framework were also used for the subcoding of the general categories. New subcategories were added as they emerged from the actions. Data collection and research was subdivided into (a) Data collection actions and (b) Research actions. Quality and accessibility of health services was subdivided into (a) Communication actions (b) Information actions (c) Trainings and direct involvement of migrants (d) Adapting services (e) Training for professionals (f) Partnerships and coordination (g) Administrative procedures (h) Identification of health needs.

The results were gathered and presented in both tables and a narrative synthesis format.

## Results

In total, 17 policy documents had been selected for analysis. The results of the policy analysis are presented in accordance with the dimensions of analysis. Integration-related and health-related plans at the national level were analysed for Portugal, Ireland and Spain. As the autonomous communities of Spain also have competency in health, two of them were selected and their plans analysed.

### Overview of the policies

The main objective of the policies, listed in Table 1, for all 3 countries and the regions of Spain is to improve the health status of the immigrant populations, based on both principles of equity and equality. Specific provisions targeting immigrants are present in either the integration or the health plan of Spain and Portugal. Ireland takes migrants into consideration with special actions in a multitude of policy plans, all gathered into the Intercultural Health Strategy.

For *Portugal*, most of the provisions targeting migrants’ health can be found in the integration plan [18]. However, if the health strategy does not provide any objective of action targeting specifically *imigrantes*, a “Health in all policies” framework is adopted and the emphasis is given on social determinants of health, cooperation between the different institutions and levels, health literacy, and interculturality. A comprehensive analysis of health and access to health services inequalities is performed and the reasons identified are similar to common migrants issues [19]. The integration plan proposes several measures specifically related to health.

In *Spain*, at the national level, health actions targeting *inmigrantes* are principally defined in the immigration plan [20]. The principles are based on the equalities on right to health regarding nationals [21]. The quality plan for the health system does not include any specific migrant policy. However, similarly to the Portuguese plan, it includes strategies that aim at improving equity in healthcare [22].

The integration plan focus on accessibility and mentions two general objectives: guaranteeing the right to health for foreigners and improving the diversity management capacity of health centres [20]. The policies from the autonomous regions follow the general direction given by the state but with more concrete actions. In Andalusia, the health plan just gives an overview of the objectives but without any action or recommendation on how to reach them. It proposes the elimination of the barriers for a true equity in accessing health services, policies in public health that aim at improving the life and work conditions of migrants and the provision of culturally adapted primary care services [23]. The integration plan, on the other hand, sets several objectives such as the improvement of the management of diversity, of access to health services and of quality accompanied by more concrete actions [24]. Improving the accessibility and knowledge of the Andalusian health system for migrants and their relatives is a priority. For Madrid, the health plan set a special programme for attention to migrants, while other actions targeting them can also be found in other parts of the plan, where a special focus on vulnerable populations seems to be important, such as for sexual and reproductive health [25]. The health plan targets migrants with a special programme of promotion of health for vulnerable groups in the immigrant population. It aims at contributing to guarantee mechanisms, actions and interventions in health enabling the adaptation of primary care to the specific needs of this population. The mental health policy also had specific actions directed towards migrants, to favour access to mental health care services [26]. In the integration plan, only a few actions are directly related to health [27]. They focus on both knowledge of the migrants on health services and health issues and the knowledge of health professionals on migration.

In *Ireland*, the primary care operational plan contains general provision on inequalities that can be applied to migrants as well as actions specifically targeting them. The overall objective of the plan is to enhance the provision of primary care services to vulnerable and disadvantaged groups through community action with the aim to reduce health inequalities. A particular emphasis is given on travellers, Roma, asylum seekers and refugees with the program for intercultural health [28]. The Intercultural Health Strategy is the most relevant document, with objectives such as improving the access to health services and the delivery, maternal health, mental health, community development, and human resources and organizational management [29]. Other plans are relevant to migrants such as the National Health Strategy “Quality and Fairness” [30] which includes strong commitments to reducing health inequalities and developing a people centred, quality and accountable health service, or the National action plan against racism [31]. The Equal Status Acts and the Social Partnership Agreements also contain provisions for a better integration and better care of cultural diversity.

### Population groups targeted

Spanish and Portuguese national policies use the words “immigrants” or “migrants” in the official language of the country but without explicitly stating who is included or excluded. They also use the term “ethnic minorities” in a few cases.

Ireland, however, takes a more detailed approach. The National Intercultural Health Strategy 2007–2012 covers migrants, asylum seekers, refugees and undocumented migrants, but also travellers, ethnic minorities, and children of migrants born in Ireland. Other plans target specific groups, especially travellers.

### Actions identified

All three countries propose a wide range of actions, as seen in Tables 2 and 3, in order to improve access and quality of health services for migrants. To achieve better adapted services for migrants, Portugal uses the “Migrant-Friendly Hospital” benchmark as well as the recommendations of the Amsterdam Declaration [18]. Spain emphasizes the fight against discrimination and racism in everyday medical practice as well as the adaptation of medical protocols to the social and cultural profiles of the populations [20]. Madrid aims at the creation of an attention to vulnerable migrants manager position in health centres, aimed at coordinating and planning activities and trainings [25]. The creation of good practices references is widely mentioned. Ireland proposes the implementation of out-of-hours general practitioner services [32], a measure easing the access for people with no job flexibility or cumulating jobs.

**Table 2:** Actions for quality and accessibility of health services

	Spain	Portugal	Ireland
Adapting services	- Promotion of equity, diversity and non- discrimination in healthcare - Inclusion of the intercultural perspective in all the health programmes - Creation of a manager position for attention of vulnerable migrants in health centres - Transfer of good practices on migration and health	- Implementation of migrant-friendly hospitals - Improve the organizational culture for migrants	- Promote a culture of diversity and non- discrimination among the administrative employees and health professionals - Implement out of hour GP services
Training for professionals	- Empowerment and trainings of health professionals on migrants’ health issues and interculturality (health determinants, intercultural communication, immigrants’ rights) - Trainings on health and interculturality for migrants, social and education workers - Trainings for interpreters and cultural mediators to work with mentally ill patients	- Trainings for health professionals and health services employees to interculturality	- Trainings for better cultural competency
Partnerships and coordination	- Partnerships and collaboration with NGOs - Creation of forums at the local level gathering relevant actors to discuss about relevant issues and responses	- Partnership with other NGOS, public and private services, and migrants to promote access to health services	- Partnerships with other institutions and NGOs targeting migrants
Communication	- Cultural Mediation programmes - Availability of translation and interpreting services, tele-translation		- Implementation of cultural mediators - Availability of interpreting services
Information	- Empower and inform migrants on health services on arrival - Translated (in various languages) material on health services, health issues, and rights and obligations.	- Informing migrants on their rights and duties regarding the NHS - Providing migrant-friendly information on health services and health issues - Training and education to fight the lack of health literacy	- Translated information material on legal entitlements and right - Translated information material on health services
Direct involvement of migrants	- Participation of migrants in the policy setting	- Integrating migrants with a medicine background into the health services - Involvement of migrants in the implementation of actions - Targeting young migrants to integrate them in the health system	- Providing support to community initiatives - Employ staff from diverse backgrounds
Administrative procedure		- Institutionalisation of procedures to facilitate PALOP migrants to access health services	
Identification of health needs	- Introduction of questions on clinical history and context - Importance of social determinants of health

**Table 3:** Actions for data collection and research

	Spain	Portugal	Ireland
Data collection	- Improvement of data collection on health and migration	None	- Collection of data on ethnicity, language and health inequalities - Develop a database on minority ethnic health - Partnerships to synergize the collection
Research	- Promotion of research - Working groups to develop strategies for health and migration issues - Making use of the results of the existing studies	None	- Research on aspect of interculturalism relevant to health needs - Conduction of Health Impact Assessment that take into account interculturality

The training of health professionals in cultural competencies and multiculturalism is a specific target for all three countries.

All three countries emphasise partnership actions putting migrants first. Their direct involvement is considered crucial for the success of the actions. Portugal and Ireland specifically mention the aim to attract health care staff with an immigrant background [29].

They also all plan partnerships with NGOs and other public and private organisations. Both Spanish regions go further with the creation of forums and commission at the local level, gathering professionals from health, social and education services as well as neighbourhood associations, NGOs, migrants associations, policy makers, youth centres to help facilitation and promotion of intrasectoral and interinstitutional coordination [24, 25].

In the area of communication, only Ireland and Spain and its regions propose the implementation of cultural mediation programmes and the availability of interpreting and translation services, with systems of tele-translation for Andalusia [20, 24, 29]. Regarding information, again only Ireland and Spain and its regions propose to distribute translated information material on legal entitlements and rights and duties on health services and on their functioning [20, 24, 25, 29], while Portugal only mentions « migrant-friendly » information [18]. Portugal and Spain both propose education initiatives to improve health literacy.

For research, as seen in Table 3, the Madrid plan recommends to make use of the existing data and to improve the diffusion of the results of the existing studies. [25].

Portugal and Ireland target specific migrant populations for specific issues. In Ireland, the Primary care operational plan aims at implementing actions specifically for Roma and travellers such as the “Roll out the asthma” education programme and cooperation with the “Diabetes Clinical programme” [28]. In Portugal, a specific category of migrants is targeted with the proposal for the institutionalization of procedures designed to improve the management of health agreements and facilitation access by migrants to health services. The target population include patients evacuated from African Countries with Portuguese as an official Language (PALOP); countries with whom Portugal has cooperation agreements in healthcare [18].

In each country, a focus is given to particular issues as seen in Table 4. Ireland sets special actions targeting minority ethnic women, as well as an expert advisory group for maternity services, emphasising more appropriate and culturally responsive services in the area [29]. Andalusia emphasises on paying attention to social determinants of health through better coordination between diverse actors, to prevent gender or child violence, xenophobia, and slavery [24].

**Table 4:** Health issues specifically addressed

	Spain	Portugal	Ireland
Health issues specifically addressed	- Sexual and reproductive health - Mental health - Paediatric services - Communicable diseases - Drug users	- Mental health	- Women’s health - Mental health - Care needs of children, families and older people - Disability - Sexual health - Alcohol and addiction

Mental health is a particular concern for the 3 countries as seen in Table 4. In Ireland, actions are being implemented for mental health services to be provided in a culturally sensitive manner and to support community initiatives aimed at providing care and support [29]. Cooperation with the national office for suicide prevention is also planned [28]. Madrid even set provision for migrants in the general mental health policy, such as the collaboration with interpreters and cultural mediators trained to work with mentally ill patients as well as extending the use of phone translation as an alternative. The identification of the issues faced by migrants in accessing mental health services also has to be extended, as well as research on transcultural mental health [26].

## Discussion

### A promising development of inclusive policies

Overall, only Ireland with its focus on “intercultural healthcare” proposes a comprehensive policy specifically targeting migrants’ health. It is also the only country which targets equally both ethnic minorities and newly arrived migrants while Portugal and Spain only focus on the latter. The differences in the focus are usually related to the different patterns and levels of immigration, so a focus on the last type of foreign population is understandable since all countries experienced large scale immigration only recently [33]. As Ireland policy also covers ethnic minorities, its policies can be considered as highly inclusive. Indeed, settled migrants and their descendants face different types of health issues than newly arrived migrants, so policies targeting them might soon be necessary in both Portugal and Spain. The focus on reproductive health and children in Spain shows that the country is moving in that direction.

The 3 countries propose the development of programmes to improve the knowledge of migrants on health services but also to a certain extent, on health issues and health literacy. Studies have suggested that this is an appropriate course of action as migrants benefit from better information on health services and entitlements, as well as from education programmes to improve health literacy [33]. However, doing so through printed material and medias as suggested might not be the best strategy. A study conducted by Scheppers et al. reports that it is preferable to make direct personal contact with the patients and their relatives and not to rely too much on other forms of communication. It indeed seems that they only discourage the ethnic minority patients from finding out more about health services [34].

Difficulties in communication, notably due to language, are an important barrier and Ireland and Spain respond to this issue by using interpreters. The use of official interpreters is therefore perceived as a high-quality service as it allows more privacy than when a family member or acquaintance is asked to help with the translation. However, its implementation is rather difficult since for certain uncommon languages it might require planning in advance and therefore might not be available for emergency situations. It is also most likely to happen that the interpreters and the patients are from the same community, a situation that can potentially introduce a bias. [35]. Nonetheless, the ambivalence introduced by the presence of a third party also happens with professional interpreters. It is always a potential barrier to effective communication and relationships between the patient and the health professional as it can, in a conscious or unconscious way, interfere with the therapeutic relationship. Bilingual professionals might appear as a solution to avoid those drawbacks, but the proficiency in the language needs to be high, and the range of languages spoken is not likely to cover all the idioms needed. [36].

Nevertheless, communication problems are not only due to language issues but are also influenced by socioeconomic and cultural factors [16]. To overcome related issues, Spain and Ireland respond by introducing cultural mediators but without specifically defining their roles. Usually, cultural mediators are health workers not only providing interpreting services but also mediating actively between health practitioners and services users. Their role is quite extensive, involving helping health professionals and patients to understand their respective points of view and advising on how to solve the problems encountered [36].

A complementary approach to cultural mediators is the empowerment on cultural matters of health professionals, an action presented by the three countries. This action directly targets providers of health services and its success hinges on their willingness to engage in it. A Belgian study provides part of the answer, concluding that health professionals do not consider that it is their responsibility to adapt to the culture but the responsibility of the patient [37]. Therefore, if they do not feel a responsibility to adapt, they are less likely to be involved in culturally competent health care [36]. Nevertheless, Portugal has initiated a rather new project in Europe consisting in actively hiring doctors with a migrant background, a measure representing a serious attempt to diversify care from within.

That having been said, truly improving access to health care and surpassing cultural and social barriers can perhaps only be achieved through the empowerment of immigrants. This path seems to be opened by all three countries since they all propose actions to include them in the policy or implementation process. Using the community resources to increase awareness and health literacy may be viewed as fundamental as it leads to overcome most of the barriers to access and participation, using appropriate communication strategies, working with cultural factors which influence behavioural change and assessing affiliation with cultural norms [38]. Still, this requires in-depth knowledge of the groups and established links with key organizations.

### Current challenges

The similarity between the countries when it comes to the type of actions could be related to the similar migration history that the countries share. However, some studies concluded that the integration model chosen by each country had more influence for promoting specific health policies than the migration history [10]. The three countries discussed here indeed fall under the multicultural model [39], a model that recognizes the specific needs of immigrants and formulate specific policies for them.

Nevertheless, other perspectives on integration exist, such as assimilationist countries that expect immigrants to use the health system without introducing any change. As a matter of example, the current debate in Austria is not about adapting the healthcare system to migrants, but rather about their integration and assimilation and their financial contribution. In Denmark, translated material is being removed and mediator services suppressed as well as the free interpreter services previously available for migrants living in the country for more than 7 years [7]. Even if those examples could be used to argue about the relativity of what can be considered to be an appropriate approach to integration, there are serious doubts about the usefulness of those policies from a public health perspective. Moreover, making healthcare less accessible in order to motivate migrants to learn the language and adapt themselves, beyond legal and ethical considerations, is a rather counterproductive policy, as health is itself a factor of integration [40]. Some findings support this idea, proving that migrants report better health in multicultural countries than in assimilationist and exclusionist countries, where inequalities between immigrants and natives were also the highest [39].

While the policy formulation is already a challenge, the real proof comes with implementation. With the sensitive nature of any public policy linked to migration in most of the European countries [11] particularily in light of the economic crisis, migrants’ right to adapted healthcare services might become more and more controversial. Indeed, public spending on health is under increasing pressure and even more so with the emergence of far-right parties that often take advantage of the widespread erroneous belief that the provision of healthcare is attracting immigrants to Europe [33].

Additionally, numerous factors such as the functioning of the health system, changing political leaders, demographic patterns of immigration, data availability, and collaboration with other organisation impact implementation.

But if the costs of such services are most often mentioned, the cost of not providing these services is generally overlooked: the human and financial costs of mistakes, misunderstandings and ineffective health care delivery might be important [41].

The adoption of national policies may therefore not be felt on the ground, a concern even more important due to the scarcity of data on implementation for the policies assessed. This lack of information makes it also impossible to assess the extent to which the experience of migrants on the ground is affected by the presence or absence of a government policy [41].

However, an absence of government policy does not necessarily mean unadapted health services for immigrants. Many projects have been implemented by health service providers, social health insurance funds, NGOs, research centres and local authorities but weren’t fully reflected in national policies. This complicates policy analysis on migration health in general, as well as the fact that policy on paper might end up having little relation to the reality. Some argue that in that sense, the lack of policies might be advantageous since a heavily regulated system can reduce the scope for individual and non-governmental actions [42].

### The role of the European Union

As attested by the recent events, immigrants are not only a concern for countries but also an European one; so is migrants’ health. The European Union’s role has historically been mainly confined to that of agreeing on common policies, strategies and specific measures to be adopted by country governments to preventing and controlling diseases [43], with the overall objective to protect European citizens. Today, at the European level, there seems to be a growing attention paid to migrants’ health as attested by the recent developments of funded research, conferences, and recommendations. Those developments have begun to find echoes in country policies, with the use of the benchmark of the “Migrant-friendly hospital project” as a key example [44] and the recommendation of the Amsterdam declaration in the Portuguese policy documents.

Still, these actions have a limited and not consistent impact all over the Union, notably in the current context of a growing public concern towards immigration. As seen above, even the willingness to develop a multicultural model of integration does not mean that it finds echo on the ground or even at the policy level. Portugal is a perfect example, since its strong involvement in the fight against migrants’ related health inequalities can be seen at the European level while it barely echoes at the country level. The MIPEX results for Portugal confirm that, while it ranks 2nd out of 38 countries in migrants’ integration, the score for health policies is relatively poor and far below the scores of Spain and Ireland [45].

The European Union must therefore take a leadership role and not only ensure that migrants’ health becomes a priority in every government’s agenda [43] but also monitor and support the implementation. Such tools as population health indexes need to be further developed to ease the monitoring of health status and needs as well as enable the comparisons between countries. Not only is there a need of migrants for demographic and economic reasons due to the ageing of the European population [43] but Europe cannot claim to be founded on the values of equality, pluralism, non-discrimination, tolerance and solidarity if it keeps a blind eye on this issue.

### Limitations

The list of policy documents identified for this analysis is not exhaustive and more recent plans might have been published during the submission process of this article. Other plans might also not have been identified during the research stage. As a consequence, it cannot be assumed that an action or policy not listed by this study is not being implemented in a given country. It should also be kept in mind that an absence of government policy does not mean that an issue is not being addressed or that the action does not exist since other institutions and organisations who take a proactive role in implementing actions.

The implementation status has not been addressed by this study. As a result, it cannot be assumed that an action identified by this study is being implemented.

## Conclusion

The findings presented here demonstrate that Ireland, Spain and Portugal have established a comprehensive set of inclusive policies for migrants’ health and can inspire other countries for migrants’ health policy development. Although only a small number of policy documents have been reviewed, there are few references to implementation, and monitoring and evaluation actions are therefore recommended. The need for monitoring and evaluation are even more important not only for the implementation but also the development of policies targeting migrants’ health which are under threat due to the financial strains on health systems and the growing aversion towards immigration and migrant communities.

However, it is fundamental to reinforce the public health perspective of migrants’ health regardless of politics or ideological views and the European Union has a crucial role in that. Although only health protection and health threats fall under the direct competence of the European Union and the capacity to foster and reinforce accessibility, quality and equity to care for migrants is a competence and responsibility of national governments, the European Union should take more proactive leadership to enforce policy making based on good practices, going clearly beyond the current recommendations.

Achieving full health potential does not depend only on health services. Many other factors and actors impact migrants’ health and have an influence on inequalities and policy makers should ensure that health is given priority across all the sectors with a role to play in improving health status. The focus laid on health policies should not underestimate that social determinants of health are the main factors causing ill-health and only a comprehensive set of policies targeting both the social causes of ill-health such as poverty, racism and education, and the health care services will lead to the necessary change.

European countries should appreciate the potential that healthcare systems have to become one of the major bridges between the immigrant populations and a welcoming society. The health system has the potential to be a powerful tool of social integration tool that can reduce social inequalities in health through the access and availability of a quality health attention to immigrant populations.
